# Supplementary material for: Altered microRNA Transcriptome in Cultured Human Liver Cells upon Infection with Ebola Virus
Source: Int J Mol Sci. 2021 Apr 6;22(7):3792. doi: 10.3390/ijms22073792 (PMC8038836; doi:10.3390/ijms22073792)
Supplement: Supplementary file 1 [file ijms-22-03792-s001.zip › Supplementary_File/C_ GO_Analysis_Results/16-30nt_go_Mayinga-24h-Huh7_vs_Control-24h-Huh7_up.mature_mirna_targets/CC_Pvalue_tree.pdf]

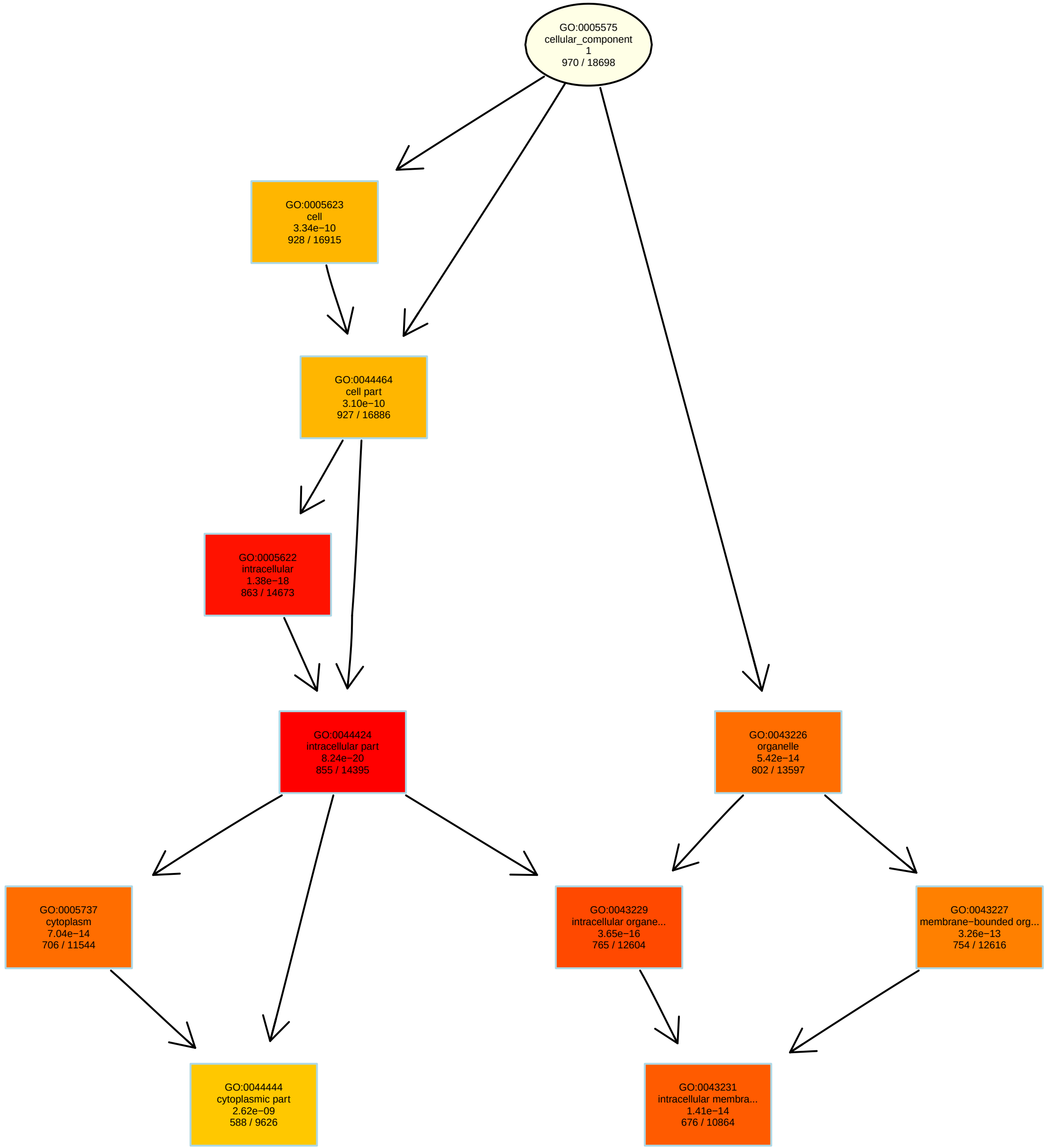
GO:0005575
cellular_component
1
970 / 18698
GO:0005623
cell
3.34e−10
928 / 16915
GO:0044464
cell part
3.10e−10
927 / 16886
GO:0005622
intracellular
1.38e−18
863 / 14673
GO:0044424
intracellular part
8.24e−20
855 / 14395
GO:0043226
organelle
5.42e−14
802 / 13597
GO:0005737
cytoplasm
7.04e−14
706 / 11544
GO:0043229
intracellular organe...
3.65e−16
765 / 12604
GO:0043227
membrane−bounded org...
3.26e−13
754 / 12616
GO:0044444
cytoplasmic part
2.62e−09
588 / 9626
GO:0043231
intracellular membra...
1.41e−14
676 / 10864
